# Genetic Variants in *FBN-1* and Risk for Thoracic Aortic Aneurysm and Dissection

**DOI:** 10.1371/journal.pone.0091437

**Published:** 2014-04-17

**Authors:** Olga A. Iakoubova, Carmen H. Tong, Charles M. Rowland, May M. Luke, Veronica E. Garcia, Joseph J. Catanese, Remo M. Moomiaie, Peter Sotonyi, Gyorgy Ascady, Demitrios Nikas, Panagiotis Dedelias, Maryann Tranquilli, John A. Elefteriades

**Affiliations:** 1 Celera-A Division of Quest Diagnostics, Alameda, California, United States of America; 2 Yale University, New Haven, Connecticut, United States of America; 3 Semmelweis University, Budapest, Hungary; 4 Athens Medical Center, Athens, Greece; 5 Evangelismos Hospital, Athens, Greece; Innsbruck Medical University, Austria

## Abstract

**Objectives:**

A recent genome wide association study (GWAS) by LeMaire et al. found that two single nucleotide polymorphisms (SNPs), rs2118181 and rs10519177 in the *FBN-1* gene (encoding Fibrillin-1), were associated with thoracic aortic dissection (TAD), non-dissecting thoracic aortic aneurysm (TAA), and thoracic aortic aneurysm or dissection (TAAD); the largest effect was observed for the association of rs2118181 with TAD. We investigated whether rs2118181 and rs10519177 were associated with TAD, TAA, and TAAD in the Yale study.

**Methods:**

The genotypes of rs2118181 and rs10519177 were determined for participants in the Yale study: 637 TAAD cases (140 TAD, 497 TAA) and 275 controls from the United States, Hungary, and Greece. The association of the genotypes with TAD, TAA and TAAD were assessed using logistic regression models adjusted for sex, age, study center and hypertension.

**Results and Conclusions:**

In the Yale study, rs2118181 was associated with TAD: compared with non-carriers, carriers of the risk allele had an unadjusted odds ratio for TAD of 1.80 (95% CI 1.15–2.80) and they had odds ratio for TAD of 1.87 (95% CI 1.09–3.20) after adjusting for sex, age, study center and hypertension. We did not find significant differences in aortic size, a potential confounder for TAD, between rs2118181 risk variant carriers and non-carriers: mean aortic size was 5.56 (95% CI: 5.37–5.73) for risk variant carriers (CC+CT) and was 5.48 (95% CI: 5.36–5.61) for noncarriers (TT) (p = 0.56). rs2118181 was not associated with TAA or TAAD. rs10519177 was not associated with TAD, TAA, or TAAD in the Yale study. Thus, the Yale study provided further support for the association of the *FBN-1* rs2118181SNP with TAD.

## Introduction

Thoracic aortic dissection (TAD), without surgical treatment, is an event that is frequently fatal [1]. The related condition of thoracic aortic aneurysm (TAA) is an important risk factor for TAD [2]. TAA develop largely asymptomatically with most patients feeling no pain or other symptoms until rupture or dissection occurs [3]. Although non-dissecting TAA patients who have undergone appropriate elective surgical treatment have a near-normal prognosis [4]–[6], and despite progress in predicting the risk of TAD in patients with established TAA based on aortic size criteria, there is a need for improvement in the assessment of risk for TAD, to better guide surgical decision making [7]. This is especially true because acute aortic dissection may occur without substantial aorta enlargement. Aortic dissection is overlooked initially in up to 40% of cases, and the mortality rate for untreated dissection approaches 1% per hour during the first 48 hours [1]. While radiographic diagnosis by ECHO or CT scan is highly accurate, it is not considered appropriate or cost-effective to screen the general population radiographically [8]. Therefore, better identification and risk assessment for TAD and TAA are essential goals. Genetic polymorphisms, in conjunction with traditional risk factors, may pinpoint individuals at greater risk for TAD or identify non-dissecting TAA better than traditional risk factors alone [9]–[13]. This is especially important for those patients at high risk for TAD who cannot be identified by aortic size alone. Thus, genetic polymorphisms have the potential to improve clinical care and reduce TAD and TAA related death [9].

A recent genome wide association study (GWAS) found that two single nucleotide polymorphisms (SNPs, rs2118181 and rs10519177) in the *FBN-1* gene were associated with TAD, TAA, and thoracic aortic aneurysm or dissection (TAAD). The largest effect was observed for the association of rs2118181 with TAD [9], [14]. *FBN-1* gene encodes for Fibrillin-1, an extracellular matrix protein, which is an essential component of the elastic fibers in the aortic wall [10]. Interestingly, heterozygosity for rare mutations in the same *FBN-1* gene causes Marfan syndrome, an autosomal dominant disorder in which patients present with TAAD in addition to ocular and skeletal phenotypes [15].

Therefore, we investigated whether rs2118181 and rs10519177 in the *FBN-1* were also associated with TAD, TAA, and TAAD in a large group of patients studied at Yale University.

## Materials and Methods

### Study Subjects

Study subjects included 140 thoracic aortic dissection cases, 497 non-dissecting thoracic aortic aneurysm cases, and 275 disease-free controls collected in the U.S. (Yale University), Hungary (Semmelweis University), and Greece (Athens Medical Center and Evangelismos Hospital). Controls (n = 275) included randomly selected unaffected individuals from Greek and Hungarian study centers who generally shared the same ethnic background, geographic location, and environmental milieu (n = 206) and unaffected spouses of patients with TAAD collected at Yale University (n = 69) who generally shared the same ethnic background, geographic location, and environmental milieu. The physicians caring for the patients verified the aneurysm and/or dissection phenotype from their own radiographic and surgical records, and provided the demographic and clinical data on the patients and controls.

The clinical characteristics of cases and controls are presented in Table 1. All subjects provided written informed consent to participate in the study; the study and informed consent procedure were approved by institutional review boards of the participating centers: the Yale University Human Investigation Committee, the Semmelweis University (Budapest, Hungary) Institutional Review Board, the Athens Hospital Center (Athens, Greece) Institutional Review Board and the Evangelismos General Hospital (Athens, Greece) Institutional Review Board.

**Table 1:** Characteristics of Dissection, Non-dissecting Aneurysm Cases, and Controls.

Characteristics	Dissection (n = 140)	Non-dissecting aneurysm (n = 497)	Control (n = 275)	p Value*	p Value†
Age, yrs	63±13.1	64±13.8	57±15.2	0.001	<0.0001
Male, n (%)	85 (60.7)	344(69.2)	107(39.2)	<0.0001	<0.0001
Smoking status, n (%)	69 (49.3)	185 (37.3)	91 (35.7)	0.01	0.69
Hypertension, n (%)	113 (81.3)	352 (71.0)	98 (38.0)	<0.0001	<0.0001
Center, n (%)				<0.0001	<0.0001
US (Yale)	100 (71.4)	347 (69.8)	69 (25.1)		
Hungary	28 (20.0)	86 (17.3)	111 (40.4)		
Greece	12 (8.6)	64 (12.9)	95 (34.6)		

*P value for Dissection cases vs. controls;

† P value for Non-dissecting aneurysm cases vs. controls.

Data presented as mean ± standard deviation for age and as a number (%) of subjects for other variables.

*P* values are from Fisher exact test, except those for age, which are from the Wilcoxon rank sum test.

### Genotyping and Laboratory Measurements

Genotypes for individual DNA samples were determined by real time kinetic polymerase chain reaction (PCR) as described previously [16].

### Statistical Analysis

Differences in traditional risk factors between TAD cases and controls, or between TAA cases and controls, were assessed by the Fisher exact test or the Wilcoxon rank sum test for discrete and continuous characteristics, respectively. An exact test was used to assess deviation of *FBN-1* genotype frequencies from Hardy-Weinberg expectations. The association of *FBN-1* risk genotypes with TAAD was assessed using logistic regression models: an unadjusted model, a model adjusted for sex and study center and that adjusted for sex, study center, age, hypertension, and smoking. Multinomial logistic regression was applied to compute odds ratios (OR), 95% confidence intervals (CI), and p-values for association between the risk genotypes or risk variant carrier status with either TAD or non-dissecting TAA versus controls.

All probability values are 2-sided and 95% confidence intervals (CI) are presented. All analyses were performed using SAS version 9.2.

## Results

### Subject Characteristics, Power, and Allele Frequencies of the *FBN-1* SNPs

The characteristics of TAD and TAA cases and controls are presented in Table 1. As might be expected, traditional risk factors such as older age, hypertension, and male gender were significantly more prevalent among cases. The smoking status differed between TAD cases and controls but not between TAA cases and controls.

Based on the observed allele frequency and risk estimates from LeMaire et al, and given the Yale Study sample size, the power to detect *FBN-1* variants association in the Yale study was >92% for rs2118181 and >82% for rs10519177.

The minor allele frequency in controls was 12.7% for rs2118181 and 26.5% for rs10519177 which were essentially identical to those observed by LeMaire et al (13% and 27%, respectively). Among controls, the rs2118181 genotype frequencies were 1.8% for CC, 21.8% for CT, 76.4% for TT, and 23.6% for carriers of the C variant; the rs10519177 genotype frequencies were 7.6% for GG, 37.8% for GA, 54.6% for AA, and 45.5% for carriers of the G variant. The genotype distribution of *FBN-1* variants among controls did not deviate from Hardy-Weinberg equilibrium expectations (p>0.64).

### Association of *FBN-1* SNPs with TAD TAA or TAAD in the Yale Study

Carriers of the rs2118181 risk variant (C allele) were at increased risk for TAD, compared with noncarriers: the odds ratio (OR) was 1.80 (95% Confidence Interval (CI) 1.15–2.80) (Table 2). Since the majority of cases were from the US (Yale University) and the majority of controls were from Hungary and Greece, and since about 23% of controls were spouses of the TAAD patients, there was a potential for confounding in this study by study center and sex. However, risk estimate for association of rs2118181 with TAD was essentially unchanged after adjusting for study center and sex: OR was 1.76, 95% CI 1.09–2.85 (Table 2). After further adjusting for age, hypertension, and smoking, the ORs for the risk of TAAD in rs2118181 risk carriers compared with noncarriers was 1.87 (95% CI 1.09–3.20) (Table 2). rs10519177 was not associated with TAD either before or after adjustment (Table 2). Neither SNP was associated with TAA, either descending or ascending TAA, or TAAD (Table 3, Table 4, and Table S1 in File S1 and Table S2 in File S1). Since the samples were collected in three different centers, we reported risk estimates and genotype counts for association of rs2118181 and rs10519177 with TAAD according to the study center (Table S3 in File S1).

**Table 2:** Association of two SNPs in *FBN-1* with Thoracic Aortic Dissection.

				Unadjusted			Adjusted*			Adjusted†	
Genotype	Case	Control	OR	95%CI	p Value	OR	95%CI	p Value	OR	95%CI	p Value
**rs2118181**											
CC	4	5	1.87	0.49–7.11	0.3605	1.12	0.26–4.81	0.8840	2.36	0.40–13.96	0.3441
CT	46	60	1.79	1.13–2.83	0.0125	1.82	1.11–2.99	0.0172	1.84	1.06–3.19	0.0314
CC + CT	50	65	1.80	1.15–2.80	0.0097	1.76	1.09–2.85	0.0211	1.87	1.09–3.20	0.0235
TT	90	210	Ref			Ref			Ref		
Additive			1.64	1.11–2.42	0.0130	1.56	1.02–2.38	0.0425	1.74	1.07–2.82	0.0254
**rs10519177**											
GG	14	21	1.39	0.67–2.89	0.3793	1.07	0.49–2.33	0.8652	1.47	0.59–3.68	0.4091
AG	54	104	1.08	0.70–1.67	0.7218	1.04	0.65–1.66	0.8685	0.86	0.52–1.43	0.5581
GG + AG	68	125	1.13	0.75–1.70	0.5473	1.05	0.68–1.62	0.8365	0.94	0.58–1.53	0.8060
AA	72	150	Ref			Ref			Ref		
Additive			1.14	0.83–1.56	0.4122	1.04	0.74–1.45	0.8233	1.04	0.72–1.52	0.8273

*Adjusted for sex and study center.

† Adjusted for sex, study center, age, hypertension, and smoking.

**Table 3:** Association of two SNPs in *FBN-1* with Non-dissecting Thoracic Aortic Aneurysm.

				Unadjusted			Adjusted*			Adjusted†	
Genotype	Case	Control	OR	95%CI	p Value	OR	95%CI	p Value	OR	95%CI	p Value
**rs2118181**											
CC	12	5	1.42	0.49–4.09	0.5159	0.80	0.24–2.70	0.7238	1.70	0.36–8.08	0.5045
CT	127	60	1.25	0.88–1.78	0.2097	1.25	0.84–1.87	0.2765	1.32	0.83–2.08	0.2421
CC + CT	139	65	1.27	0.90–1.78	0.1758	1.21	0.82–1.79	0.3361	1.34	0.86–2.09	0.2030
TT	355	210	Ref			Ref			Ref		
Additive			1.23	0.91–1.68	0.1713	1.15	0.81–1.64	0.4380	1.32	0.88–1.98	0.1869
**rs10519177**											
GG	42	21	1.15	0.66–2.02	0.6171	0.88	0.47–1.64	0.6869	1.25	0.59–2.69	0.5614
AG	192	104	1.07	0.78–1.46	0.6920	1.02	0.72–1.46	0.9010	0.88	0.59–1.32	0.5310
GG + AG	234	125	1.08	0.80–1.45	0.6102	1.00	0.71–1.40	0.9775	0.93	0.64–1.37	0.7154
AA	260	150	Ref			Ref			Ref		
Additive			1.07	0.85–1.35	0.5657	0.97	0.75–1.26	0.8315	1.00	0.74–1.35	0.9914

*Adjusted for sex and study center.

† Adjusted for sex, study center, age, hypertension, and smoking.

**Table 4:** Association of the SNPs in *FBN-1* with Thoracic Aortic Dissection or Aneurysm.

				Unadjusted			Adjusted*			Adjusted†	
Genotype	Case	Control	OR	95%CI	p Value	OR	95%CI	p Value	OR	95%CI	p Value
**rs2118181**											
CC	16	5	1.51	0.55–4.18	0.4273	0.87	0.27–2.81	0.8112	1.83	0.39–8.47	0.4430
CT	173	60	1.36	0.97–1.91	0.0728	1.37	0.93–2.01	0.1131	1.42	0.91–2.23	0.1229
CC + CT	189	65	1.37	0.99–1.90	0.0573	1.32	0.91–1.93	0.1439	1.45	0.93–2.24	0.0982
TT	445	210	Ref			Ref			Ref		
Additive			1.32	0.99–1.77	0.0611	1.24	0.88–1.74	0.2227	1.41	0.94–2.10	0.0947
**rs10519177**											
GG	56	21	1.21	0.70–2.06	0.4967	0.92	0.51–1.68	0.7919	1.30	0.62–2.75	0.4914
AG	246	104	1.07	0.79–1.44	0.6638	1.03	0.73–1.45	0.8800	0.88	0.59–1.30	0.5195
GG + AG	302	125	1.09	0.82–1.45	0.5454	1.01	0.73–1.40	0.9661	0.94	0.65–1.36	0.7350
AA	332	150	Ref			Ref			Ref		
Additive			1.09	0.87–1.35	0.4689	0.99	0.77–1.27	0.9209	1.01	0.76–1.36	0.9317

*Adjusted for sex and study center.

† Adjusted for sex, study center, age, hypertension, and smoking.

Aortic size is a risk factor for TAD and could also potentially confound the observed association between rs2118181 *FBN-1* SNPs and TAD. Since we could not adjust the association of the SNPs with TAD by aortic size because aortic size was not available for aneurysm-free controls, we investigated the association between rs2118181 and aortic size among TAD cases. We did not find significant differences in aortic size according to rs2118181 risk variant carrier status: mean aortic size was 5.56 (95% CI: 5.37–5.73) for risk variant carriers (CC+CT) and was 5.48 (95% CI: 5.36–5.61) for noncarriers (TT) (p = 0.56).

## Discussion

We found that the *FBN-1* rs2118181 risk variant (C), a variant that has been previously reported to be associated with TAD in a GWAS conducted by LeMaire et al. [14] is also associated with TAD in the Yale Study: carriers of the rs2118181 risk variant were at 76% greater risk for TAD than non-carriers. The effect size for this association remained essentially unchanged after adjusting for potential confounders: sex, age, study center and hypertension. This association of the *FBN-1* rs2118181 risk variant with TAD was also unlikely to be confounded by aortic size because rs2118181 variant was not associated with aortic size among TAD cases. In contrast, another *FBN-1* risk variant, rs10519177, was not associated with TAD in the Yale study. Neither rs2118181 nor rs10519177 were associated with either non-dissecting TAA or TAAD in the Yale study. However, given the strong and consistent association of rs10519177 observed in the LeMaire et al. studies, the negative finding for this SNP in the current study could be due to the play of chance and should be further investigated. Interestingly, LeMaire et al, also observed a stronger association of the *FBN-1* rs2118181 risk variant with TAD than with non-dissecting thoracic aortic aneurysm [14].

We and others have previously proposed aortic size criteria for surgical intervention for TAD [17]. These criteria permit extirpation of the abnormal thoracic aorta before rupture and/or dissection are likely to occur. However, some TAD events occur before these size criteria are met [7], [18]–[19]. Adding the *FBN-1* risk variant may provide an opportunity to enhance our TAD risk predictive models that currently include aortic size and other clinical risk factors. by identifying among patients with moderately large aneurysm those who are at greater risk for TAD due to the presence of the *FBN-1* risk variant.

There are some limitations of this study. This genetic study had a case-control design and therefore the subjects who died of thoracic aortic dissection or non-dissecting aneurysm rupture could not be included in the analysis. The absence of fatal cases in case-control studies can have a substantial negative impact on the association results [20]. Because the case-control study design precluded obtaining follow-up information on controls, it is possible that some controls may experience thoracic aortic dissection or non-dissecting aneurysm rupture later in life. However, since thoracic aortic dissection or non-dissecting aneurysm is relatively rare, the number of misclassified controls is likely to be small. Matching controls were not collected for all subjects and thus the matching could not be fully exploited in analyses. In addition, the control group recruited at Yale University was heterogeneous and smaller than the case group. Because there were only a few non-Caucasian participants in this study, we analyzed only Caucasians; therefore, the association of the *FBN-1* risk variants with TAD, TAA, and TAAD should be further investigated in studies that would include other ethnic groups.

## Conclusions

In the multicenter case-control Yale Study, *FBN-1* rs2118181 was associated with TAD but not with TAA or TAAD; rs10519177 was not associated with TAD, TAA, or TAAD. The negative finding for the rs10519177 SNP in the current study could be due to chance and should be further investigated. Thus, the study reported herein provided further support for the association of the *FBN-1* rs2118181 SNP with TAD.

## Supporting Information

File S1 (DOCX)
