# Supplementary material for: Genetic Variants in FBN-1 and Risk for Thoracic Aortic Aneurysm and Dissection
Source: PLoS One. 2014 Apr 17;9(4):e91437. doi: 10.1371/journal.pone.0091437 (PMC3990573; doi:10.1371/journal.pone.0091437)
Supplement: File S1 — (DOCX) [file pone.0091437.s001.docx]

Table S1. Association of *FBN-1* SNPs with Ascending Non-dissecting Thoracic Aortic Aneurysm

|  |  |  |  | | Unadjusted |  |  | |  | | Adjusted* |  |  | |  | Adjusted** |  |
| --- | --- | --- | --- | --- | --- | --- | --- | --- | --- | --- | --- | --- | --- | --- | --- | --- | --- |
| Genotype | Case | Control | OR | | 95%CI | p Value |  | | OR | | 95%CI | p Value |  | | OR | 95%CI | p Value |
| rs2118181 |  |  |  | |  |  |  | |  | |  |  |  | |  |  |  |
| CC | 11 | 5 | 1.80 | | 0.62-5.28 | 0.2808 |  | | 1.19 | | 0.32-4.39 | 0.7918 |  | | 2.16 | 0.42-11.04 | 0.3556 |
| CT | 97 | 60 | 1.33 | | 0.92-1.92 | 0.1349 |  | | 1.34 | | 0.85-2.12 | 0.2046 |  | | 1.33 | 0.80-2.22 | 0.2702 |
| CC + CT | 108 | 65 | 1.36 | | 0.95-1.95 | 0.0898 |  | | 1.33 | | 0.86-2.06 | 0.2053 |  | | 1.38 | 0.84-2.27 | 0.2028 |
| TT | 256 | 210 | Ref | |  |  |  | | Ref | |  |  |  | | Ref |  |  |
| Additive |  |  | 1.33 | | 0.97-1.83 | 0.0758 |  | | 1.26 | | 0.85-1.86 | 0.2451 |  | | 1.37 | 0.88-2.14 | 0.1685 |
|  |  |  |  | |  |  |  | |  | |  |  |  | |  |  |  |
| rs10519177 |  |  |  | |  |  |  | |  | |  |  |  | |  |  |  |
| GG | 30 | 21 | 1.12 | | 0.62-2.04 | 0.7066 |  | | 0.98 | | 0.65-1.48 | 0.9303 |  | | 1.25 | 0.53-2.93 | 0.6109 |
| AG | 143 | 104 | 1.08 | | 0.78-1.50 | 0.6492 |  | | 0.92 | | 0.45-1.87 | 0.8177 |  | | 0.87 | 0.55-1.37 | 0.5355 |
| GG + AG | 173 | 125 | 1.09 | | 0.79-1.49 | 0.6032 |  | | 0.97 | | 0.66-1.3 | 0.8773 |  | | 0.92 | 0.60-1.42 | 0.7056 |
| AA | 191 | 150 | Ref | |  |  |  | | Ref | |  |  |  | | Ref |  |  |
| Additive |  |  | 1.07 | | 0.84-1.37 | 0.6005 |  | | 0.97 | | 0.75-1.33 | 0.8319 |  | | 1.00 | 0.71-1.40 | 0.9796 |
|  |  |  |  | |  |  |  | |  | |  |  |  | |  |  |  |
|  |  |  |  |  | | | |  | |  | | | |  |  |  |  |

*Adjusted for sex and study center

**Adjusted for sex, study center, age, hypertension, and smoking

Table S2. Association of *FBN-1* SNPs with Descending Non-dissecting Thoracic Aortic Aneurysm

|  |  |  |  | Unadjusted |  |  |  | Adjusted* |  |  |  | Adjusted** |  |
| --- | --- | --- | --- | --- | --- | --- | --- | --- | --- | --- | --- | --- | --- |
| Genotype | Case | Control | OR | 95%CI | p Value |  | OR | 95%CI | p Value |  | OR | 95%CI | p Value |
| rs2118181 |  |  |  |  |  |  |  |  |  |  |  |  |  |
| CC | 1 | 5 | 0.52 | 0.06-4.51 | 0.5516 |  | 0.56 | 0.05-5.96 | 0.6327 |  | 1.21 | 0.01-267.3 | 0.9437 |
| CT | 22 | 60 | 0.95 | 0.55-1.65 | 0.8572 |  | 0.94 | 0.51-1.72 | 0.8320 |  | 0.85 | 0.41-1.77 | 0.6713 |
| CC + CT | 23 | 65 | 0.92 | 0.53-1.58 | 0.7544 |  | 0.91 | 0.50-1.65 | 0.7581 |  | 0.86 | 0.42-1.78 | 0.6797 |
| TT | 81 | 210 | Ref |  |  |  | Ref |  |  |  | Ref |  |  |
| Additive |  |  | 0.90 | 0.55-1.47 | 0.6585 |  | 0.89 | 0.52-1.54 | 0.6884 |  | 0.87 | 0.43-1.76 | 0.6946 |
|  |  |  |  |  |  |  |  |  |  |  |  |  |  |
| rs10519177 |  |  |  |  |  |  |  |  |  |  |  |  |  |
| GG | 10 | 21 | 1.25 | 0.56-2.82 | 0.5862 |  | 1.05 | 0.43-2.56 | 0.9094 |  | 1.53 | 0.44-5.25 | 0.5028 |
| AG | 37 | 104 | 0.94 | 0.58-1.52 | 0.7894 |  | 0.89 | 0.52-1.52 | 0.6669 |  | 0.63 | 0.33-1.21 | 0.1670 |
| GG + AG | 47 | 125 | 0.99 | 0.63-1.56 | 0.5862 |  | 0.92 | 0.56-1.52 | 0.7428 |  | 0.72 | 0.39-1.34 | 0.3041 |
| AA | 57 | 150 | Ref |  |  |  | Ref |  |  |  | Ref |  |  |
| Additive |  |  | 1.04 | 0.74-1.48 | 0.8164 |  | 0.97 | 0.66-1.42 | 0.8803 |  | 0.90 | 0.55-1.48 | 0.6845 |
|  |  |  |  |  |  |  |  |  |  |  |  |  |  |

*Adjusted for sex and study center

**Adjusted for sex, study center, age, hypertension, and smoking

Table S3. Association of the SNPs in *FBN-1* with Thoracic Aortic Dissection or Aneurysm According to Study Center

|  |  |  |  |  |  |  |  |
| --- | --- | --- | --- | --- | --- | --- | --- |
| Study Center | Genotype |  | Case | Control | OR | 95%CI | p Value |
| rs2118181 |  |  |  |  |  |  |  |
| US (Yale) | CC + CT |  | 131 | 20 | 1.03 | 0.59-1.76 | 0.9301 |
|  | TT |  | 313 | 49 | Ref |  |  |
| Hungary | CC + CT |  | 30 | 23 | 1.37 | 0.74-2.54 | 0.3237 |
|  | TT |  | 84 | 88 | Ref |  |  |
| Greece | CC + CT |  | 28 | 22 | 1.94 | 0.99-3.77 | 0.0522 |
|  | TT |  | 48 | 73 | Ref |  |  |
|  |  |  |  |  |  |  |  |
| rs10519177 |  |  |  |  |  |  |  |
| US (Yale) | GG + AG |  | 212 | 37 | 0.79 | 0.48-1.31 | 0.3643 |
|  | AA |  | 232 | 32 | Ref |  |  |
| Hungary | GG + AG |  | 54 | 50 | 1.10 | 0.65-1.86 | 0.7268 |
|  | AA |  | 60 | 61 | Ref |  |  |
| Greece | GG + AG |  | 36 | 38 | 1.35 | 0.73-2.48 | 0.3344 |
|  | AA |  | 40 | 57 | Ref |  |  |
|  |  |  |  |  |  |  |  |
